# Association of Different Types of Childhood Maltreatment With Emotional Responding and Response Control Among Youths

**DOI:** 10.1001/jamanetworkopen.2019.4604

**Published:** 2019-05-24

**Authors:** Karina S. Blair, Joseph Aloi, Kathleen Crum, Harma Meffert, Stuart F. White, Brittany K. Taylor, Emily K. Leiker, Laura C. Thornton, Patrick M. Tyler, Niraj Shah, Kimberly Johnson, Heba Abdel-Rahim, Jennie Lukoff, Matthew Dobbertin, Kayla Pope, Seth Pollak, R. James Blair

**Affiliations:** 1 Center for Neurobehavioral Research, Boys Town National Research Hospital, Boys Town, Nebraska; 2 Department of Psychiatry, University of Nebraska Medical Center, Omaha; 3 Department of Psychiatry & Behavioral Sciences, Medical University of South Carolina, Charleston; 4 Target Holding, Groningen, the Netherlands; 5 Department of Neurological Sciences, University of Nebraska Medical Center, Omaha; 6 Department of Psychiatry, Creighton University, Omaha, Nebraska; 7 Department of Psychology, University of Wisconsin, Madison

## Abstract

**Importance:**

Childhood maltreatment is associated with serious developmental consequences that may be different depending on the form of maltreatment. However, relatively little research has investigated this issue despite implications for understanding the development of psychiatric disorders after maltreatment.

**Objective:**

To determine the association of childhood maltreatment and potential differential associations of childhood abuse or neglect with neural responsiveness within regions of the brain implicated in emotional responding and response control.

**Design, Setting, and Participants:**

In this cross-sectional study, participants aged 10 to 18 years with varying levels of prior maltreatment as indexed by the Childhood Trauma Questionnaire (CTQ) were recruited from a residential care facility and the surrounding community. Blood oxygen level–dependent response data were analyzed via 2 analyses of covariance that examined 2 (sex) × 3 (task condition [view, congruent, incongruent]) × 3 (valence [negative, neutral, positive]) with Blom-transformed covariates: (1) total CTQ score; and (2) abuse and neglect subscores. Data were collected from April 1, 2016, to June 30, 2018. Data analyses occurred from June 10, 2018, to October 31, 2018.

**Main Outcomes and Measures:**

Blood oxygenation level–dependent signals in response to an Affective Stroop task were measured via functional magnetic resonance imaging.

**Results:**

The sample included 116 youths (mean [SD] age, 15.0 [2.2] years; 70 [60.3%] male). Fifteen participants reported no prior maltreatment. The remaining 101 participants (87.1%) reported at least some prior maltreatment, and 55 (54.5%) reported significant maltreatment, ie, total CTQ scores were greater than the validated CTQ score threshold of 40. There were significant total CTQ score × task condition associations within the bilateral postcentral gyrus, left precentral gyrus, midcingulate cortex, middle temporal gyrus, and superior temporal gyrus (left postcentral gyrus: *F* = 11.73; partial η^2^ = 0.14; right postcentral and precentral gyrus: *F* = 9.81; partial η^2^ = 0.10; midcingulate cortex: *F* = 12.76; partial η^2^ = 0.12; middle temporal gyrus: *F* = 13.24; partial η^2^ = 0.10; superior temporal gyrus: *F* = 10.33; partial η^2^ = 0.11). In all examined regions of the brain, increased maltreatment was associated with decreased differential responsiveness to incongruent task trials compared with view trials (left postcentral gyrus: *r* = −0.34; 95% CI, −0.17 to −0.51; right postcentral and precentral gyrus: *r* = −0.31; 95% CI, −0.14 to −0.49; midcingulate cortex: *r* = −0.36; 95% CI, −0.18 to −0.53; middle temporal gyrus: *r* = −0.35; 95% CI, −0.17 to −0.52; superior temporal gyrus: *r* = −0.37; 95% CI, −0.20 to −0.55). These interactions were particularly associated with level of abuse rather than neglect. A second analysis of covariance revealed significant abuse × task condition (but not neglect × task) interactions within the midcingulate cortex (*F* = 13.96; partial η^2^ = 0.11), right postcentral gyrus and inferior parietal lobule (*F* = 15.21; partial η^2^ = 0.12), left postcentral and precentral gyri (*F* = 11.16; partial η^2^ = 0.12), and rostromedial frontal cortex (*F* = 10.36; partial η^2^ = 0.08)). In all examined regions of the brain, increased abuse was associated with decreased differential responsiveness to incongruent task trials compared with view trials (midcingulate cortex: partial *r* = −0.33; *P* < .001; right postcentral gyrus and inferior parietal lobule: partial *r* = −0.41; *P* < .001; left postcentral and precentral gyri: partial *r* = −0.40; *P* < .001; and rostromedial frontal cortex: partial *r* = −0.40; *P* < .001).

**Conclusions and Relevance:**

These data document associations of different forms of childhood maltreatment with atypical neural response. This suggests that forms of maltreatment may differentially influence the development of psychiatric pathology.

## Key Points

**Question:**

Are the amount and type (abuse vs neglect) of childhood maltreatment differentially associated with the responsiveness of regions of the brain implicated in emotional responding and response control?

**Findings:**

In this cross-sectional study including 116 youths aged 10 to 18 years, the amount of childhood maltreatment was inversely associated with the responsiveness of regions of the brain involved in response control and positively associated with emotional responding. This association was also found specifically for abuse but not for neglect.

**Meaning:**

Different types of childhood maltreatment may have different associations with atypical neural responses; therefore, they may have different associations with different forms of psychiatric neurobiology.

## Teaser

This cross-sectional study examines the association of amount and different type of childhood maltreatment with responsiveness in regions of the brain implicated in emotional responding and response control in youths.

## Introduction

Childhood maltreatment is associated with neurodevelopmental disruption,^1^ psychopathology^2,3,4,5^ (ie, heightened threat sensitivity^6,7,8,9^), heightened amygdala responsiveness,^10,11,12^ and disrupted reinforcement-based decision making.^13,14,15^ Maltreatment may also be associated with executive dysfunction, although there is some inconsistency in the findings.^16,17,18,19^ Two studies examining response control reported increased responsiveness within the dorsal cingulate cortex and midcingulate cortex and precentral and postcentral gyri during response control in maltreated children and adolescents.^16,17^ However, 2 other studies using similar tasks reported that a history of childhood maltreatment in women^18^ and a history of exposure to childhood stress in adults^19^ were associated with decreased responsiveness in the frontal or frontal-parietal regions of the brain. Moreover, a 2016 study^20^ reported a history of childhood maltreatment was associated with significantly reduced activation during sustained attention within regions of the brain in adults, including the left inferior cortex, dorsolateral prefrontal cortex, insula, and temporal cortex, compared with healthy controls.

This inconsistency in the association of maltreatment with executive function might reflect differences in the forms of maltreatment experienced by participants. To our knowledge, much of the previous literature has either grouped together participants who have experienced different forms of maltreatment^11,21^ or only considered 1 form of maltreatment, eg, deprivation.^10^ However, different forms of childhood maltreatment may have distinct consequences for development (even if many individuals who have experienced maltreatment experienced multiple forms of maltreatment).^22,23,24,25,26,27^ In particular, the literature suggests that threatening contexts (eg, physical abuse [PA] or sexual abuse [SA]) increase threat responsiveness while deprivation (eg, physical neglect [PN] or emotional neglect [EN]) disrupts aspects of learning, memory, and executive function.^22,26,27^

The goal of this study was to examine the association of childhood maltreatment and childhood abuse or neglect with the responsiveness of neural regions implicated in emotional responding and response control via the Affective Stroop task.^28^ We chose an emotion-based response control task rather than one that was affectively neutral because of previous research indicating that maltreatment disrupts emotional regulation, which might in turn disrupt response control or other forms of executive function.^28,29,30^ In the Affective Stroop task, participants either perform a goal-directed activity (ie, counting the number of numerals) in the context of emotional and neutral distracter images (eFigure, A and B, in the Supplement) or simply view the emotional and neutral images (eFigure, C, in the Supplement). The task indexes systems engaged in response control via the main effect of task condition. Regions of the brain implicated in response control or the organization of motor responses show increased responses to number task trials compared with view trials (eg, dorsomedial frontal cortex and lateral frontal lobe, anterior insula, parietal lobe, premotor cortex, and motor cortex). In contrast, regions of the brain implicated in distracter representation show increased responses to view trials compared with task trials (eg, temporal cortex, ventromedial frontal cortex, rostromedial frontal cortex [rmFC], and amygdala). Impaired goal-directed emotional regulation manifests as reduced suppression of activity within regions of the brain involved in distracter representation on number task trials.^28,29,30,31,32,33,34^

Therefore, we hypothesized first that maltreatment would be positively associated with responsiveness to distracters and inversely associated with the responsiveness of regions of the brain involved in response control. Second, we hypothesized that abusive forms of maltreatment (ie, SA, emotional abuse [EA], and PA) and neglectful forms of maltreatment (ie, EN and PN) would differ in their associations with atypical neural functioning.^23^ Specifically, on the basis of previous suggestions,^22^ we hypothesized that abuse would be positively associated with responsiveness to distracters while neglect would be negatively associated with the responsiveness of regions of the brain involved in response control. In addition to these hypotheses, we conducted exploratory analyses to examine the extent to which different subforms of abuse and neglect might be associated in different ways with the responsiveness of regions of the brain implicated in emotional responding and response control. Given the exploratory nature of this last goal, we did not make specific predictions a priori.

## Methods

### Participants

Participants included 116 youths aged 10 to 18 years (Table 1). Participants were recruited either within 1 week of their arrival at a residential care facility (n = 70) or from the surrounding community (n = 46). Participants recruited from the care facility had been referred for behavioral and mental health problems. Participants from the community were recruited through flyers.

**Table 1.** Associations of Maltreatment and Forms of Maltreatment With Demographic Variables and Diagnosis Information

Variable	Mean (SD) [Range]			*F* Test for Female vs Male	*P* Value
	All (N = 116)	Female (n = 46)	Male (n = 70)		
IQ	103.1 (12.2) [77-134]	102.4 (12.4) [77-133]	103.6 (12.2) [80-134]	0.24	.62
Age, y	15.0 (2.2) [10-18]	14.9 (2.3) [10-18]	15.1 (2.0) [10-18]	0.18	.68
CTQ score					
Total	36.3 (14.4) [25-93]	41.0 (18.8) [25-93]	33.2 (9.6) [25-65]	8.68	.004
Emotional abuse	8.2 (4.1) [5-25]	9.4 (5.1) [5-25]	7.5 (3.0) [5-17]	6.36	.01
Physical abuse	6.7 (3.4) [5-23]	7.1 (3.7) [5-23]	6.5 (3.1) [5-21]	0.99	.32
Sexual abuse	6.4 (4.6) [5-25]	8.5 (6.8) [5-25]	5.1 (0.5) [5-9]	17.98	<.001
Emotional neglect	8.1 (4.1) [5-22]	8.6 (4.6) [5-20]	7.8 (3.8) [5-22]	0.93	.34
Physical neglect	6.7 (2.7) [5-21]	7.2 (3.5) [5-21]	6.3 (2.0) [5-14]	2.56	.11

Abbreviation: CTQ, Childhood Trauma Questionnaire.

Clinical characterizations were performed through psychiatric interviews by 2 authors who are licensed and board-certified child and adolescent psychiatrists (M.D. and K.P.) with the participants and separately with their parents or guardians to adhere closely to common clinical practice. Written informed consent from the parents or guardians and written assent from the participants were obtained. In all cases, youths had the right to decline participation at any time before or during the study. Consent documents were reviewed with the parents or legal guardians and written permission was obtained at the initial visit for community participants or at the time of intake for youths placed in Boys Town programs (eAppendix 1 in the Supplement). Assent was obtained from the youth from the community in a separate session. The Boys Town National Research Hospital institutional review board approved this study.

Data for this study were collected from April 1, 2016, to June 30, 2018. Data analysis occurred from June 10, 2018, to October 31, 2018. This study is reported following the Strengthening the Reporting of Observational Studies in Epidemiology (STROBE) reporting guideline.

### Childhood Trauma Questionnaire

Child maltreatment was assessed using the Childhood Trauma Questionnaire (CTQ), a 28-item self-reported measure containing 5 subscales indexing EA, PA, SA, EN, and PN. The CTQ has excellent psychometric properties, including internal consistency, test-retest reliability, and convergent and discriminant validity with interviews and clinician reports of maltreatment.^35^ Individuals respond to each item using a 5-point Likert scale, and scores range from 25 (no history of abuse or neglect) to 125 (extreme abuse or neglect).

### Functional Magnetic Resonance Imaging Task

The Affective Stroop task was adapted from a 2007 study by Blair et al^28^ and a 2016 study by Hwang et al^32^ (eAppendix 2 and eFigure in the Supplement). In brief, on congruent and incongruent trials, participants responded using buttons to indicate the number of numerals (3, 4, 5, or 6) presented temporally between picture images (eFigure in the Supplement). In view trials, participants received a blank between the display of the picture images (no response required). Sixteen images for each affect (negative, neutral, and positive) were selected from the International Affective Picture System.^36^

Each participant underwent 2 rounds of testing. Each round included 16 presentations of each valence × condition combination throughout the round. In addition, forty 2500-millisecond fixation points were randomly presented throughout each round. Thus, each participant was presented with a total of 32 trials of each valence × task condition.

### Functional Magnetic Resonance Imaging Parameters

Whole-brain blood oxygen level–dependent functional magnetic resonance imaging data were acquired using a MAGNETOM Skyra magnetic resonance scanner (Siemens Medical Solutions) (eAppendix 3 in the Supplement). Data were analyzed with a random-effects general linear model using Analysis of Functional NeuroImages functional magnetic resonance imaging software (National Institute of Mental Health Scientific and Statistical Computing Core).^37^

Ten regressors depicting each of the response types were created: negative view, negative congruent, negative incongruent, neutral view, neutral congruent, neutral incongruent, positive view, positive congruent, positive incongruent, and error or missed responses. Voxelwise group analyses involved transforming single-subject β coefficients into the 3-dimensional standard coordinate system of Talairach space.^38^ Generalized linear model fitting was performed with the 10 regressors, 6 motion regressors (rotation around the inferior-superior axis, rotation around the right-left axis, rotation around the anterior-posterior axis, displacement in the superior direction, displacement in the left direction, and displacement in the posterior direction), and a regressor modeling a first-order baseline drift function.

### Statistical Analysis

To reduce skewness and kurtosis, a Blom transformation was applied to participants’ total CTQ and CTQ subscale scores. Posttransformation, skewness and kurtosis values for CTQ and all subscores were between −1 and 1.

#### Clinical Correlations

Pearson correlation analyses were conducted to determine the associations among Blom-transformed CTQ total scores, abuse (EA + SA + PA) score, neglect (EN + PN) score, age, IQ score, sex, and whether the individual had received 1 or more of 6 psychiatric diagnoses (conduct disorder, attention-deficit/hyperactivity disorder, major depressive disorder, generalized anxiety disorder, social anxiety disorder, or posttraumatic stress disorder) or not (scored 1 or 0, respectively). Steiger *z *tests were performed to determine whether there were significant differences in correlation strengths between amount of abuse or neglect and any of the psychiatric diagnoses. For all these analyses, *P* values were 2-tailed and considered significant at less than .05.

#### Blood Oxygen Level–Dependent Response Data

Two core analyses of covariance (ANCOVAs) were performed on the blood oxygen level–dependent response data. Both had the same basic model structure. Specifically, because of sex differences in total CTQ scores and EA and SA subscores, full 2 (sex) × 3 (task condition [view, congruent, incongruent]) × 3 (valence [negative, neutral, positive]) ANCOVAs were conducted.

The first ANCOVA, focusing on the association of maltreatment with atypical brain function, involved 1 covariate, total CTQ score. The second ANCOVA, focusing on differential associations of abuse compared with neglect with atypical neural functioning, involved 2 covariates, abuse (EA + PA + SA) subscore and neglect (EN + PN) subscore.

Correction for multiple comparisons was performed using 3dClustSim, a spatial clustering operation in the Analysis of Functional NeuroImages software, using the autocorrelation function (−acf) with 10 000 Monte Carlo simulations for the whole-brain analysis. The initial threshold for *P* value was set at .001. This process resulted in an extant *k* threshold of 24 voxels for the whole brain (multiple comparison–corrected, *P* < .05). To facilitate future meta-analytic work, effect sizes (partial η^2^) for all clusters are reported in Table 2.

**Table 2.** Significant Areas of Activation From the Analysis Involving the CTQ Total Score

Region	Brodmann Area	Voxels	Coordinates			*F* Value	Partial η^2^
			x	y	z		
Total CTQ score × task condition							
R midcingulate cortex	31/6	51	7.5	−22.5	47.5	12.76	0.12
R postcentral gyrus	3	59	40.5	−25.5	56.5	9.81	0.10
L postcentral and precentral gyri and premotor cortex	43	59	−52.5	−7.5	17.5	11.73	0.14
L middle TG	21	35	−55.5	−13.5	−6.5	13.24	0.10
L superior TG	22	26	−43.5	−25.5	−0.5	10.33	0.11
L declive culmen		76	−13.5	−58.5	−12.5	13.76	0.12
Abuse vs neglect							
Total CTQ abuse × task condition							
L rostromedial frontal cortex^a^	9	54	−16.5	49.5	26.5	10.36	0.08
R midcingulate cortex^a^	31/6	52	7.5	−22.5	47.5	13.96	0.11
R postcentral gyrus and inferior parietal lobule^b^	40	235	31.5	−37.5	53.5	15.21	0.12
L precentral and postcentral gyri and premotor cortex^b^	6	109	−49.5	−7.5	23.5	11.16	0.12
R precentral and postcentral gyri^a^	3	30	61.5	−10.5	23.5	13.30	0.10
Total CTQ neglect × task condition							
R cuneus	18	62	7.5	−76.5	17.5	10.28	0.09

Abbreviations: CTQ, Childhood Trauma Questionnaire; L, left; R, right; TG, temporal gyrus.

^a^ Regions of the brain showing significant abuse × task condition associations were also significant for the same association if only abuse was used as a covariate in the analysis of covariance.

^b^ Significant areas of activation involving abuse vs neglect. Activations from whole brain analyses were considered significant at *P* < .001. When corrected for multiple comparisons, analyses were considered significant at *P* < .05.

Associations of covariates with task variables identified via the ANCOVAs were interpreted via correlational analyses using SPSS statistical software version 22.0 (IBM), and significance was set at *P* less than .05. For the results of the second (2-covariate) ANCOVA, these correlations controlled for the second covariate.

Our exploratory goal—examining the extent to which different subforms of abuse are associated with different neural response—was examined by 3 exploratory 2 (sex) × 3 (task condition) × 3 (valence) ANCOVAs. Covariates were (1) EA and PA, (2) SA and all other forms of abuse combined (given the very small number of male participants reporting SA in this sample, the association with SA was examined only among the female participants), and (3) EN and PN.

## Results

### Levels of Maltreatment and Clinical Correlations

There were 116 youths included in the study (mean [SD] age, 15.0 [2.2] years; 70 [60.3%] male participants). The mean (SD) IQ score was 103.1 (12.2). Fifteen youths reported no prior maltreatment (total CTQ score = 25). The remaining 101 youths reported at least some prior maltreatment, with 55 (54.5%) reporting significant amounts of maltreatment (ie, their CTQ scores were greater than validated thresholds [total CTQ score, ≥40; EA subscore, ≥10; SA subscore, ≥8; PA subscore, ≥8; EN subscore, ≥15; or PN subscore, ≥8]).^11,36^ All participants reporting significant SA or PA were discussed by 1 of 3 of us (K.C., P.M.T., or M.D.) with the participants’ consultants to confirm that this had been previously identified and was followed up.

There were no significant associations of total CTQ score or CTQ subscore with age or IQ score. However, compared with male participants, female participants had a significantly greater mean total CTQ score (*F* = 8.68; *P* = .01), mean EA subscore (*F* = 6.36; *P* = .004), and mean SA subscore (*F* = 17.87; *P* < .001) (Table 1). In addition, there were significant positive correlations of both amount of abuse and amount of neglect with all 6 psychiatric diagnoses assessed and with the self-report measures (eTable 1 in the Supplement). There were no significant differences in correlation strengths between amount of abuse or neglect and any of the psychiatric diagnoses or self-report measures except posttraumatic stress disorder. Among patients who had received diagnoses of posttraumatic stress disorder, the amount of abuse showed a significantly greater correlation with diagnosis compared with amount of neglect (Steiger *z* = 2.62; *P* < .05).

### Association of Maltreatment With Atypical Neural Functioning

Details on the measurement and calculation of behavior and movement data can be found in eTable 4 in the Supplement. Our first ANCOVA revealed regions showing several significant interactions (Table 2).

**Figure 1.**
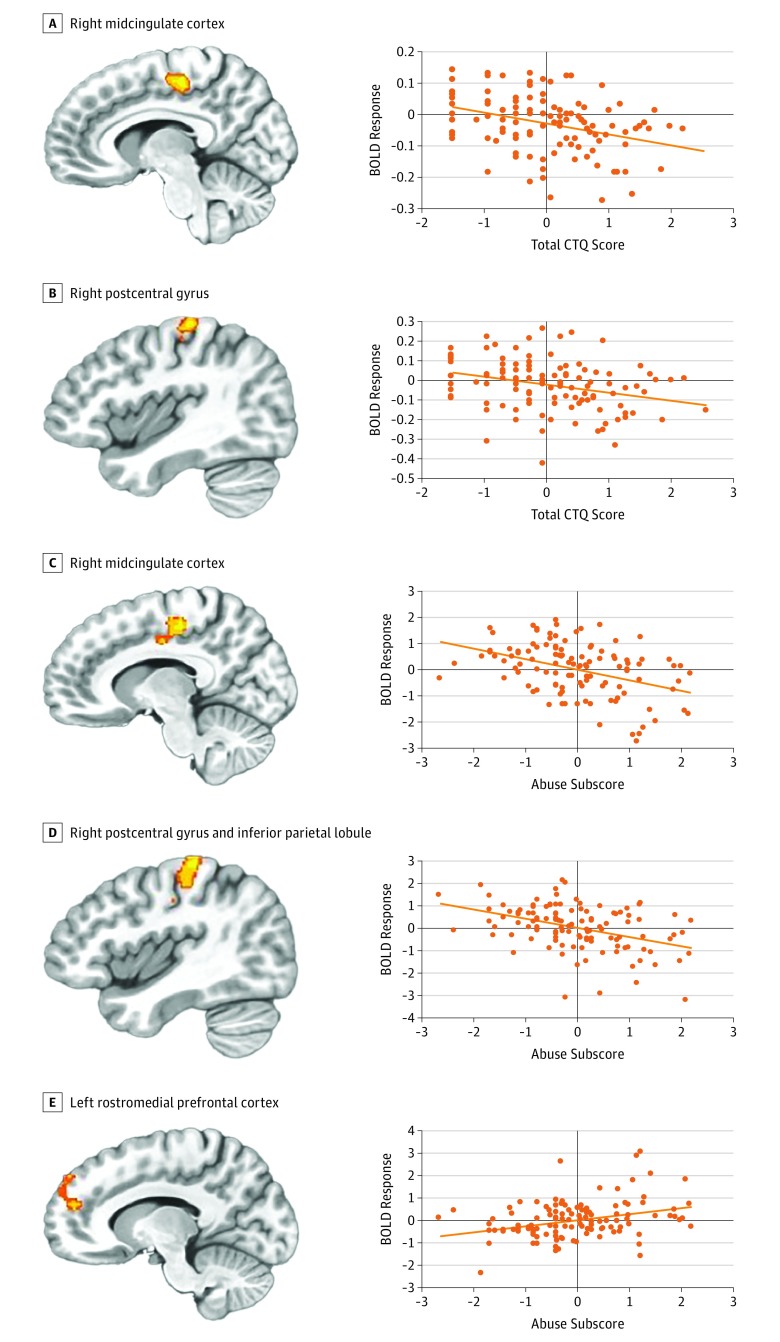
Associations of Total Child Trauma Questionnaire (CTQ) Score × Task Condition and Abuse × Task Condition A and B, Increased total CTQ scores were associated with decreased differential responsiveness to incongruent task trials compared with view trials in the right midcingulate cortex (x = 7.5; y = −22.5; z = 47.5) (A) and the right postcentral gyrus (x = 40.5; y = −25.5; z = 56.5) (B). C and D, Increased amounts of abuse were associated with decreased differential responsiveness to incongruent task trials compared with view trials in the right midcingulate cortex (x = 7.5, y = −22.5; z = 47.5) (C) and the right postcentral gyrus and inferior parietal lobule (x = 31.5; y = −37.5; z = 53.5) (D). E, Increased amounts of abuse were associated with increased differential responsiveness to view trials compared with incongruent task trials in the left rostromedial prefrontal cortex (x = −16.5; y = 49.5; z = 26.5). A-E, Scatterplots depict the correlations (A and B) and partial correlations (C-E) with adjusted residuals for each of the brain regions. Adjusted residuals for the Blom-transformed *z*-scored CTQ scores (x-axis: A and B) or abuse subscores (x-axis: C-E) are plotted against adjusted residuals for the mean blood oxygen level–dependent (BOLD) response difference in incongruent trials and view trials.

There were significant total CTQ score × task condition interactions in regions of the brain implicated in response control and motor responding (left postcentral gyrus: *F* = 11.73; partial η^2^ = 0.14; right postcentral and precentral gyri: *F* = 9.81; partial η^2^ = 0.10; midcingulate cortex: *F* = 12.76; partial η^2^ = 0.12; middle temporal gyrus [mTG]: *F* = 13.24; partial η^2^ = 0.10; sTG: *F* = 10.33; partial η^2^ = 0.11) and in regions of the brain implicated in distracter representation (left mTG, and right superior temporal gyrus [sTG]) (Table 2). Within all examined regions, increased maltreatment was associated with decreased differential responsiveness to both incongruent task trials (left postcentral gyrus: *r* = −0.34; 95% CI, −0.17 to −0.51; right postcentral and precentral gyri: *r* = −0.31; 95% CI,−0.14 to −0.49; midcingulate cortex: *r* = −0.36; 95% CI, −0.18 to −0.53; mTG: *r* = −0.35; 95% CI, −0.17 to −0.52; sTG: *r* = −0.37; 95% CI, −0.20 to −0.55) and congruent task trials (left postcentral gyrus: *r* = −0.42; 95% CI, −0.25 to −0.59; right postcentral and precentral gyri: *r* = −0.39; 95% CI, −0.22 to −0.56; midcingulate cortex: *r* = −0.38; 95% CI, −0.20 to −0.55; mTG: *r* = −0.45; 95% CI, −0.28 to −0.61; sTG: *r* = −0.43; 95% CI, −0.26 to −0.60) compared with view trials (Figure 1A and B). All other statistically significant results are presented in eTable 2 in the Supplement.

### Differential Associations of Abuse Compared with Neglect With Atypical Neural Functioning

Our second ANCOVA revealed statistically significant abuse × task condition interactions in regions of the brain implicated in response control and motor responding (eg, midcingulate cortex: *F* = 13.96; partial η^2^ = 0.11 [Figure 1C]; right postcentral gyrus and inferior parietal lobule: *F* = 15.21; partial η^2^ = 0.12 [Figure 1D]; left postcentral and precentral gyri: *F* = 11.16; partial η^2^ = 0.12) and those implicated in distracter representation that was suppressed by goal-directed activity (eg, rmFC: *F* = 10.36; partial η^2^ = 0.08) (Table 2 and Figure 1E). Within the regions of the brain implicated in response control and motor responding, after adjusting for covariates, the amount of abuse was negatively correlated with differential responsiveness to incongruent trials (midcingulate cortex: partial *r* = 0.33; *P* < .001; right postcentral gyrus and inferior parietal lobule: partial *r* = −0.41; *P* < .001; left precentral and postcentral gyri: partial *r* = −0.40; *P* < .001) and congruent trials (right inferior parietal lobule and postcentral gyrus: partial *r* = −0.37; *P* < .001) compared with view trials (Figure 1C and D). Within the rmFC, the amount of abuse was positively correlated with differential responsiveness to view relative to both incongruent trials (rmFC: partial *r* = −0.40; *P* < .001) and congruent trials (partial *r* = −0.30; *P* = .002).

There were significant neglect × task condition interactions within cuneus (Table 2). After adjusting for covariates, the amount of neglect was negatively correlated with differential responsiveness to congruent trials compared with view trials (*r* = −0.38; *P* < .001). All other significant results are presented in eTable 3 in the Supplement.

### Examining Potential Confounders of Recruitment Strategy and Diagnosis

Given that participants were recruited from both a residential treatment center and from the community, we examined whether this variable could have critically influenced our results. We repeated our 2 core ANCOVAs by adding residential care facility vs community as a covariate. This added covariate was associated with minor changes to the results reported in Table 2. eTable 4 and eTable 5 in the Supplement present the full overview of these analyses.

We examined diagnostic status as a potential confounder via a series of ANCOVAs involving an additional covariate coding each psychiatric condition (present vs not present). In all cases, the added covariate was associated with minor changes to the results reported in Table 2. eTable 4 and eTable 5 in the Supplement present the full overview of these analyses.

### Exploring Associations of Different Subforms of Abuse With Atypical Neural Functioning

#### Emotional Abuse × Task Condition Interactions

**Figure 2.**
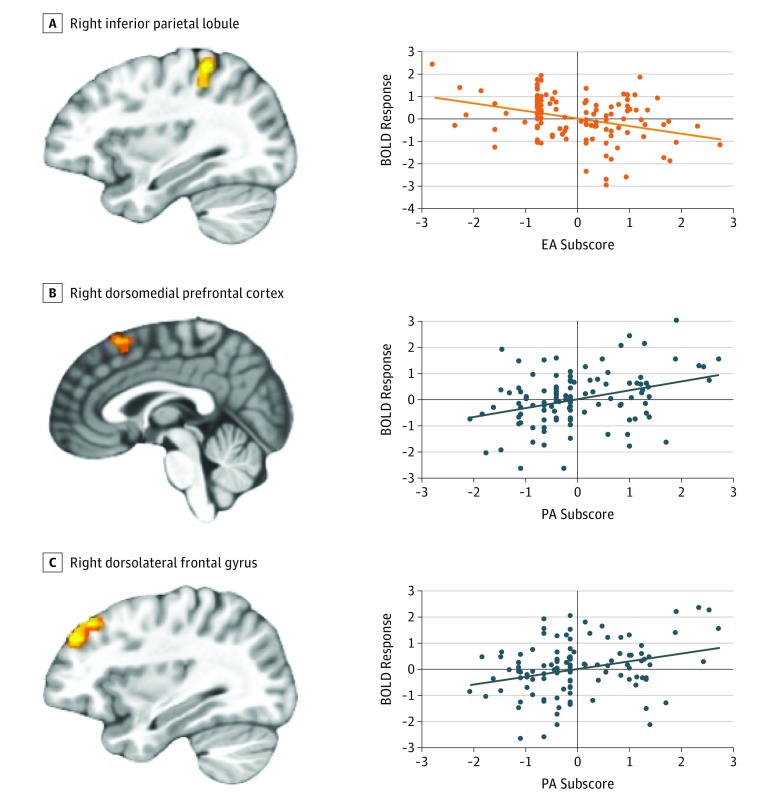
Associations of Emotional Abuse (EA) × Task Condition and Physical Abuse (PA) × Task Condition A, Increased EA scores were associated with decreased differential responsiveness to incongruent task trials compared with view trials in the right inferior parietal lobule (x = 31.5; y = −37.5; z = 53.5). B and C, Increased PA scores were associated with increased differential responsiveness to negative compared with positive trials in the right dorsomedial prefrontal cortex (x = 7.5; y = 31.5; z = 56.5) (B) and the right dorsolateral frontal gyrus (x = 31.5; y = 40.5; z = 32.5) (C). A-C, Scatterplots depict the partial correlations and adjusted residuals for each of the regions. Adjusted residuals for the Blom-transformed *z*-scored EA (x-axis: A) or PA scores (x-axis: B and C) are plotted against adjusted residuals for the mean blood oxygen level–dependent (BOLD) responses to incongruent compared with view trials (y-axis: A), and negative compared with positive trials (y-axis: B and C).

There were significant EA × task condition interactions in the inferior parietal lobule and culmen (eTable 6 in the Supplement). Within the inferior parietal lobule and culmen, amount of EA was negatively correlated with differential responsiveness to both incongruent task trials (inferior parietal lobule: partial *r* = −0.31, *P* = .001; culmen: partial *r* = −0.34, *P* < .001) and congruent task trials (inferior parietal lobule: partial *r* = −0.23, *P* = .01; culmen: partial *r* = −0.29, *P* = .002) relative to view trials (Figure 2A).

#### Physical Abuse × Valence Interactions

There were significant PA × valence associations in the dorsomedial prefrontal and lateral frontal cortices (eTable 6 in the Supplement). Within both regions, amount of PA was positively correlated with differential responsiveness to negative stimuli compared with positive stimuli (prefrontal cortex: partial *r* = 0.30, *P* = .001; lateral frontal cortex: partial *r* = 0.34, *P* < .001) (Figure 2B and C).

#### Sexual Abuse × Task Condition Interactions

**Figure 3.**
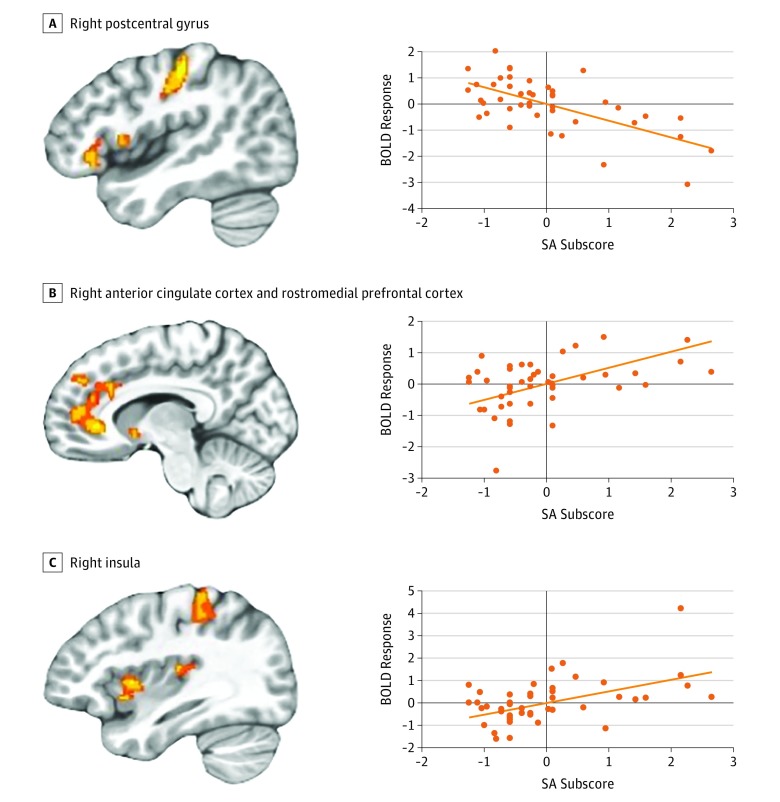
Interactions of Sexual Abuse (SA) × Task Condition A-C, Increased SA scores were associated with increased differential responsiveness to view compared with incongruent task trials in the right postcentral gyrus (x = 43.5; y = −22.5; z = 44.5) (A), the right anterior cingulate cortex and rostromedial prefrontal cortex (x = 10.5; y = 31.5; z = 2.5) (B), and the right insula (x = 34.5; y = 7.5; z = 5.5) (C). Scatterplots depict the partial correlations and adjusted residuals for each of the regions. Adjusted residuals for the Blom-transformed *z*-scored SA scores (x-axis) are plotted against adjusted residuals for the mean blood level–dependent (BOLD) responses to view compared with incongruent task trials.

There were significant SA × task condition associations in the anterior cingulate cortex, rmFC, and bilateral postcentral gyrus that overlapped with regions of the brain showing abuse × task condition associations in the main analysis (eTable 7 in the Supplement). Within all regions examined, amount of SA was positively correlated with differential responsiveness to view trials compared with incongruent task trials (anterior cingulate cortex and rmFC: partial *r* = 0.51; *P* < .001; right postcentral gyrus: partial *r* = 0.65; *P* < .001; left postcentral gyrus: partial *r* = 0.56; *P* < .001) (Figure 3). Other associations of subforms of abuse with atypical neural functioning analyses are listed in eTables 6-8 in the Supplement.

## Discussion

The goals of this study were to determine the extent to which maltreatment, abuse, and neglect may be differentially associated with atypical neural functioning. We found that maltreatment was associated with reduced responsiveness of regions of the brain implicated in response control and increased responsiveness of regions of the brain implicated in responding to and representing affective information. We also found that the amount of abuse, but not the amount of neglect, was associated with increased responsiveness of regions of the brain implicated in responding to and representing affective information (rmFC, mTG, and sTG). Additionally, and in contrast with predictions, we found that the amount of abuse, but not the amount of neglect, was associated with decreased responsiveness of regions of the brain implicated in response control and motor responding (inferior parietal lobule and postcentral gyrus). Exploratory analyses of different subforms of abuse and neglect also found an association of amount of PA with enhanced responsiveness to threat information within regions of the brain implicated in attentional processing (dorsomedial frontal cortex and lateral frontal cortex). There was also an association of SA with enhanced processing of salient visual stimuli in regions of the brain involved in the representation of emotional valence.

Additionally, we found that the amount of maltreatment was inversely associated with responsiveness of regions of the brain involved in response control. Notably, our findings related to the postcentral gyrus and midcingulate cortex are similar to 2 previous studies examining response control and behavioral inhibition in maltreated children and adolescents.^16,17^ These studies found maltreatment increased responsiveness within these regions of the brain during response inhibition, although decreased responsiveness as a function of maltreatment has also been previously reported.^18,19,20^ Potentially, maltreatment disrupts the functional efficiency of regions of the brain implicated in response control and behavioral inhibition, and the disruption may be expressed as increased or decreased responsiveness as a function of task parameters. A similar argument has been made for similarly inconsistent findings in patients with depression.^39,40^

We also found that maltreatment was positively associated with responsiveness to distracters and emotional stimuli within the rmFC, ventromedial frontal cortex, mTG, and sTG. Previous structural imaging studies have reported that these regions of the brain are smaller in individuals exposed to prior maltreatment.^41,42,43^ The rmFC and ventromedial frontal cortex have been implicated in the representation of valence^44^ and, for the rmFC in particular, affect-based self-referential processing.^45^ Therefore, our data are consistent with suggestions that the amount of maltreatment (particularly abuse) is associated with enhanced processing of salient stimuli.^6,7,8,9^

We found that amount of abuse was associated with increased responsiveness of regions of the brain implicated in responding to and representing affective information (rmFC, mTG, and sTG). Amount of neglect was also associated with a heightened responsiveness to salient visual stimuli, albeit only within visual cortices. In short, amount of maltreatment may generally increase responsiveness to salient visual stimuli. However, it is possible that abuse may increase responsiveness to affect-based stimuli (consistent with hypervigilance to threat) while neglect does so to general environmental stimuli (consistent with emotional numbing).

We found no association of amount of neglect with decreased responsiveness in regions of the brain implicated in response control and motor responding. Instead, amount of abuse was associated with decreased responsiveness in these regions (inferior parietal lobule, postcentral gyrus, and midcingulate cortex). In their 2017 article, McLaughlin and Sheridan^22^ hypothesized that cognitive deprivation disrupts aspects of learning, memory, and executive function. While our study does not support that hypothesis, it is possible that PN and EN, as indexed by the CTQ, do not equate to cognitive deprivation.^46^ Future work will require more precise indices of cognitive deprivation to test the hypothesis.

Our analysis of potential differential associations of specific forms of abuse and neglect with atypical neural functioning must be considered to be exploratory. However, there are several features of interest. First, PA was the only form of maltreatment shown to demonstrate an exaggerated response to threat on this task. This agrees with a 2000 study^23^ and a 2005 study^47^ suggesting that PA is, compared with other forms of maltreatment, particularly associated with an attentional threat bias. Second, there were notable overlaps in regions of the brain disrupted by both EA and SA, which supported the findings related to abuse generally compared with neglect. It is possible that both have profound impacts on development, perhaps by disrupting capacities to form relationships with others.

### Limitations

There are several limitations that should be noted with respect to our results. First, the forms of neglect measured by the CTQ are indirect measures of cognitive deprivation. As such, our data do not provide a direct test of the cognitive deprivation hypothesis.^22^ Second, consistent with considerable previous work,^2,3,4,48,49,50,51^ increasing amount of maltreatment was associated with increasing severity of psychopathology. Accordingly, our results might reflect psychopathology rather than maltreatment. Ameliorating this concern is the fact that there were no significant differences in correlation strengths between amount of abuse or amount of neglect with any of the psychiatric diagnoses except posttraumatic stress disorder. Further, the follow-up analyses that we conducted with psychiatric diagnosis as separate covariates for our 2 main analyses did not significantly change our results (eTable 4 and eTable 5 in the Supplement), suggesting that psychiatric diagnostic status did not significantly confound the results. Third, our study involved participants from a residential care facility and from the community, and it is possible that this recruitment strategy could have influenced our results. However, the results from follow-up analyses with recruitment strategy as a covariate were in line with the results reported without this covariate (eTable 4 and eTable 5 in the Supplement). Thus, recruitment strategy does not appear to have been a prime determinant of the results. Fourth, most participants who had experienced abuse had also experienced neglect, potentially making the associations of these different forms of maltreatment difficult to untangle. Importantly, though, the regions of the brain showing significant abuse × task associations showed these associations whether the neglect covariate was present in the ANCOVA (Table 2) or not (eTable 9 in the Supplement). Moreover, none of these revealed neglect × task associations even at very low initial thresholds (eTable 10 in the Supplement). Fourth, our sample identified very few male participants who reported experiences of SA. As such, the conclusions regarding the neural associations of SA are based on female participants only.

## Conclusions

In conclusion, this study found that amount of childhood maltreatment was inversely associated with the responsiveness of regions of the brain involved in response control and positively associated with responsiveness to emotional stimuli and distractors. This association was statistically more significant for abuse compared with neglect. Moreover, exploratory analyses suggested that SA was associated with widespread disruptions of neural systems involved in emotional responding, while PA was associated with heightened processing of threat. It is plausible that these potentially differential associations could underpin potentially differential risks of specific forms of psychiatric sequelae as functions of form and amount of abuse.

## Notes

Supplement. **eFigure.** Task Illustration **eAppendix 1.** Further Details on Consent and Assent Procedure and Exclusion Criteria **eAppendix 2.** Details on the Affective Stroop Task **eAppendix 3.** Details of Scanning Parameters **eAppendix 4.** Behavioral and Movement Data **eTable 1.** Clinical Correlations **eTable 2.** Analysis of Significant Areas of Activation for Total CTQ Score Not Reported in Table 2 **eTable 3.** Analysis of Significant Areas of Activation for Abuse vs Neglect Not Reported in Table 2 **eTable 4.** Analysis of Significant Areas of Activation for Total CTQ Score Reported in Table 2 With Added Covariates for Recruitment and Clinical Diagnoses **eTable 5.** Analysis of Significant Areas of Activation for Abuse vs Neglect Reported in Table 2 With Added Covariates for Recruitment and Clinical Diagnoses **eTable 6.** Analysis of Significant Areas of Activation for Abuse: EA vs PA **eTable 7.** Analysis of Significant Areas of Activation for SA: SA vs EA, PA, EN, and PN **eTable 8.** Analysis of Significant Areas of Activation for Neglect: EN vs PN **eTable 9.** Analysis of Significant Areas of Activation for Abuse as the Only Covariate **eTable 10.** Analysis of Significant Areas of Activation for Neglect as the Only Covariate
