# Supplementary material for: Association of Different Types of Childhood Maltreatment With Emotional Responding and Response Control Among Youths
Source: JAMA Netw Open. 2019 May 24;2(5):e194604. doi: 10.1001/jamanetworkopen.2019.4604 (PMC6632148; doi:10.1001/jamanetworkopen.2019.4604)
Supplement: Supplement. — eFigure. Task Illustration eAppendix 1. Further Details on Consent and Assent Procedure and Exclusion Criteria eAppendix 2. Details on the Affective Stroop Task eAppendix 3. Details of Scanning Parameters eAppendix 4. Behavioral and Movement Data eTable 1. Clinical Correlations eTable 2. Analysis of Significant Areas of Activation for Total CTQ Score Not Reported in Table 2 eTable 3. Analysis of Significant Areas of Activation for Abuse vs Neglect Not Reported in Table 2 eTable 4. Analysis of Significant Areas of Activation for Total CTQ Score Reported in Table 2 With Added Covariates for Recruitment and Clinical Diagnoses eTable 5. Analysis of Significant Areas of Activation for Abuse vs Neglect Reported in Table 2 With Added Covariates for Recruitment and Clinical Diagnoses eTable 6. Analysis of Significant Areas of Activation for Abuse: EA vs PA eTable 7. Analysis of Significant Areas of Activation for SA: SA vs EA, PA, EN, and PN eTable 8. Analysis of Significant Areas of Activation for Neglect: EN vs PN eTable 9. Analysis of Significant Areas of Activation for Abuse as the Only Covariate eTable 10. Analysis of Significant Areas of Activation for Neglect as the Only Covariate [file jamanetwopen-2-e194604-s001.pdf]

Supplementary Online Content

Blair KS, Aloi J, Crum K, et al. Association of different types of childhood maltreatment with emotional responding and reponse control among youths. *JAMA Netw Open*. 2019;2(5):e194604. doi:10.1001/jamanetworkopen.2019.4604.

eFigure. Task Illustration
eAppendix 1. Further Details on Consent and Assent Procedure and Exclusion Criteria
eAppendix 2. Details on the Affective Stroop Task
eAppendix 3. Details of Scanning Parameters
eAppendix 4. Behavioral and Movement Data
eTable 1. Clinical Correlations
eTable 2. Analysis of Significant Areas of Activation for Total CTQ Score Not Reported in Table 2
eTable 3. Analysis of Significant Areas of Activation for Abuse vs Neglect Not Reported in Table 2.
eTable 4. Analysis of Significant Areas of Activation for Total CTQ Score Reported in Table 2 With Added Covariates for Recruitment and Clinical Diagnoses
eTable 5. Analysis of Significant Areas of Activation for Abuse vs Neglect Reported in Table 2 With Added Covariates for Recruitment and Clinical Diagnoses
eTable 6. Analysis of Significant Areas of Activation for Abuse: EA vs PA
eTable 7. Analysis of Significant Areas of Activation for SA: SA vs EA, PA, EN, and PN
eTable 8. Analysis of Significant Area of Activation for Neglect: EN vs PN
eTable 9. Analysis of Significant Areas of Activation for Abuse as the Only Covariate
eTable 10. Analysis of Significant Areas of Activation for Neglect as the Only Covariate

This supplementary material has been provided by the authors to give readers additional information about their work.

© 2019 Blair KS et al. *JAMA Network Open.*

eFigure. Task Illustration

Example of **(a)** Negative Incongruent, **(b)** Negative Congruent, and **(c)** Negative View trial.

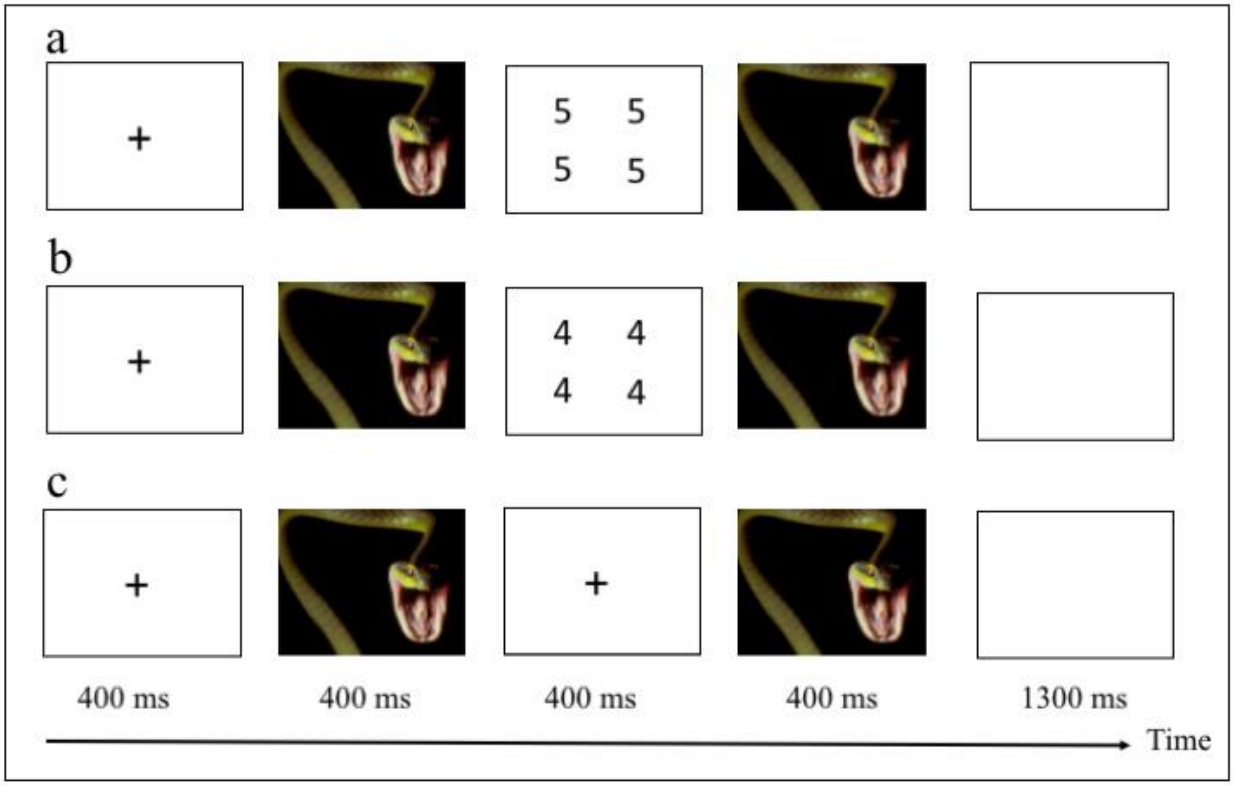

© 2019 Blair KS et al. *JAMA Network Open.*

eAppendix 1. Further Details on Consent and Assent Procedure And Exclusion Criteria

Participants

Consent and Assent. A doctoral-level researcher obtained informed consent from a parent/legal guardian. Consent was either obtained at time of admission to residential treatment, or over the phone shortly after admission. Youth were approached for assent after parental consent had been obtained, and was conducted by a member of the research team. For youth not in residential treatment, both consent and assent was obtained at the beginning of data collection. In all cases, youth have the right to decline participation at any time before or during the study. It was made clear to all participants and their parents/legal guardians that their decision with respect to participation had no influence on their clinical care.

Exclusion criteria. Exclusion criteria included IQ<80 assessed with the Wechsler Abbreviated Scale of Intelligence-II (WASI two-subtest form),[28] current psychosis, pervasive developmental disorders, Tourette's disorder, neurological disorders, pregnancy, non-psychiatric medical conditions that require the use of medication that may have psychotropic effects (e.g., beta blockers or steroids), presence of metallic objects in the body (e.g., metal plates, pacemakers), and claustrophobia. Current psychiatric conditions (other than psychotic disorders or pervasive developmental disorders) were not exclusionary. Use of psychotropic medications for psychiatric indications (e.g., stimulants, selective serotonin reuptake inhibitors) were not exclusory. However, participants on stimulant medication were asked to withhold medication on the day of the scan. Six potential participants were excluded due to MRI incompatible dental work (e.g., braces). In addition, another 6 participants refused participation on the task due to the task being too difficult/ they did not like it.

© 2019 Blair KS et al. *JAMA Network Open.*

eAppendix 2. Details on the Affective Stroop Task

The affective Stroop task was adapted from prior work by our group.[23, 31] The emotional stimuli consisted of 16 negative, 16 neutral, and 16 positive pictures selected from the International Affective Picture System (IAPS).[32] The individual cognitive task stimuli consisted of displays of numbers and the cognitive task involved deciding how many numbers were displayed in each display (see Supplemental Figure 1). Specifically, participants pressed button 3, 4, 5, or 6 to indicate whether there were 3, 4, 5, or 6 numbers in the display.

Each trial began with a fixation point presented in the middle of the screen. For trials involving a goal-directed task (*task* trials), the fixation point was replaced by an image presented for 400ms, followed by the numerical display presented for 400ms, followed by the image presented for a further 400ms, followed by a blank stimulus for 1300ms. On incongruent or difficult task trials, the Arabic numeral distracter information was inconsistent with the numerosity information (e.g., four 5s; see eFigure 1a). On congruent task trials, the Arabic numeral distracter information was consistent with the numerosity information; (e.g., four 4s; see eFigure 1b). For the view or no task trials (*view* trials; see eFigure 1c) the numerical display was simply replaced by a fixation point.

There were two runs, each consisting of 16 presentations of each Valence-by-task Condition randomized throughout the run. In addition, 40 fixation points (staying on the screen for the duration of a condition trial 2500ms) were randomly presented throughout each run. Thus, each participant was presented with 32 trials of each Valence-by-Task Condition across the two runs.

© 2019 Blair KS et al. *JAMA Network Open.*

eAppendix 3. Details of Scanning Parameters

Whole-brain blood oxygen level dependent (BOLD) fMRI data were acquired using a 3.0 Tesla Siemens Skyra Magnetic Resonance Scanner. A total of 384 functional images were taken, divided over two runs, with a T2* weighted gradient echo planar imaging (EPI) sequence (repetition time (TR)=2500ms, echo time (TE)=27ms, flip angle=90°, field-of-view (FOV)=240mm). Whole-brain coverage was obtained with 43 axial slices (thickness, 2.5mm; voxel size 2.6x2.6x2.5mm^3; distance factor 21%). In the same session, a high-resolution T1-weighed anatomical image was acquired to aid with spatial normalization (MP-RAGE, repetition time=2200ms, echo time=2.48ms; 230mm field of view; 8° flip angle; 256x208 matrix) was acquired to register with the EPI dataset. Whole-brain coverage was obtained with 176 axial slices (thickness 1mm; voxel size 0.9x0.9x1mm^3, distance factor 50%).

fMRI Analysis: Data Preprocessing and Individual Level Analysis

Functional MRI data were preprocessed and analyzed using Analysis of Functional NeuroImages (AFNI) software.[33] Data from the first four repetitions were collected prior to magnetization equilibrium and were discarded. The anatomical scan for each participant was registered to the Talairach and Tournoux atlas[34] and each participant’s functional EPI data were registered to their Talairach anatomical scan in AFNI. Functional images were motion corrected and spatially smoothed with a 6-mm full width half maximum Gaussian kernel. The data then underwent time series normalization and these results were multiplied by 100 for each voxel. Therefore, the resultant regression coefficients are representative of a percentage of signal change from the mean.

Following this, regressors depicting each of the response types were created by convolving the train of stimulus events with a gamma-variate haemodynamic response function

© 2019 Blair KS et al. *JAMA Network Open.*

to account for the slow haemodynamic response. This involved 10 regressors (Negative View, Negative Congruent, Negative Incongruent, Neutral View, Neutral Congruent, Neutral Incongruent, Positive View, Positive Congruent, Positive Incongruent, error/ missed responses). Linear regression modelling was then performed using the regressors described above plus regressors to model a first order baseline drift function. This produced for each voxel and each regressor, a beta coefficient and its associated t-statistic.

© 2019 Blair KS et al. *JAMA Network Open.*

eAppendix 4. Behavioral and Movement Data

Statistical Analyses Performed

Behavioral and movement data: The reaction time (RT) and accuracy data were analysed using two separate 2 (Sex: Male, Female) by 2 (Task Condition: Congruent, Incongruent) by 3 (Valence (Sex: Male, Female) by 2 (Task Condition: Congruent, Incongruent) by 3 (Valence: Negative, Neutral, Positive) ANCOVAs with BLOM transformed CTQ Total scores as the covariate.

Correlation analyses were conducted to determine the associations between BLOM transformed CTQ total scores, abuse (EA+SA+PA) and neglect (EN+PN) scores, and censored volumes, average motion per volume, and maximum displacement during scanning. Volumes were censored if there was >0.5 mm motion across adjacent volumes. For all these analyses significance was considered at $p<0.05$.

Results

Behavioral data

There were main effects of task for both RT and accuracy ($F(1,113)=245.71$ & 65.16, $p<0.001$ for both; $\eta p^2=0.676$ & 0.366 respectively), responses to incongruent relative to congruent task trials were slower (M[Incongruent=848.60; 95% CI, 808.78-888.42); M[Congruent]=790.10; 95% CI, 750.57-829.62), and less accurate (M[Incongruent=24.43; 95% CI, 23.31-25.54); M[Congruent]=26.40; 95% CI, 25.51-27.29). There was also a main effect of valence for RT ($F(2,226)=6.89$, $p=0.004$; $\eta p^2=0.049$); participants were slower for both negative and positive relative to neutral trials ($F(1,115)=6.31$ & 10.68 respectively; $p=0.013$ & 0.001; $\eta p^2=0.052$ & 0.085; (M[Negative=825.91; 95% CI,786.56-865.36); M[Neutral]=815.31; 95% CI,

© 2019 Blair KS et al. *JAMA Network Open.*

776.56-854.07) M[Positive]=827.56; 95% CI, 789.72-865.41). Two additional 2 (Sex) by 2 (Task Condition: Congruent, Incongruent) by 3 (Valence: Negative, Neutral, Positive) ANCOVAs were conducted on the RT and accuracy data with BLOM transformed Abuse (emotional, sexual, physical) and Neglect (emotional, physical) amount scores as the covariates. These ANOVAs revealed the same main effects of task ($F(1,112)=242.12$ & 63.87, $p<0.00$; $\eta p^2=0.369$ & 0.085, for accuracy and RT respectively) and valence (again RT only: $F(2,224)=6.95$, $p<0.001$; $\eta p^2=0.049$). However, there were no significant interactions of amount of abuse or neglect with task variables.

Movement data

There were no significant correlations between CTQ Total Score, abuse, neglect, or any of the abuse or neglect sub-scores (EA, SA, PA, EN, PN) and censored volumes (r range=0.004 to 0.082; ns), average motion per volume (r range=0.004 to 0.084; ns), and maximum displacement during scanning (r range=0.001 to 0.072; ns). No participant had >6% censored volumes.

© 2019 Blair KS et al. *JAMA Network Open.*

eTable 1. Clinical Correlations

Correlations (*r*)	CTQ: Total	CTQ: Abuse	CTQ: Neglect
CD	.365**	.313**	.338**
ADHD	.239**	.213*	.204*
MDD	.324**	.253**	.327**
GAD	.378**	.354**	.295**
SAD	.256**	.227*	.240**
PTSD	.455**	.483**	.267**

CD=Conduct Disorder; ADHD=Attentional Deficit Hyperactivity Disorder; MDD=Major Depressive Disorder; GAD=Generalized Anxiety Disorder; SAD=Social Anxiety Disorder; PTSD=Post Traumatic Stress Disorder. CTQ=Childhood Trauma Questionnaire. Abuse comprised of combined emotional, physical and sexual abuse scores. Neglect comprised of combined emotional and physical neglect scores. For correlational analysis, diagnosis coded as No Diagnosis=0, Diagnosis=1 **. Correlation is significant at the 0.001 level (2-tailed). *. Correlation is significant at the 0.05 level (2-tailed).

© 2019 Blair KS et al. *JAMA Network Open.*

eTable 2. Analysis of Significant Areas of Activation for Total CTQ Score Not Reported in Table 2

REGION	BA	Voxels	X	Y	Z	F-value
Sex						
R middle frontal gyrus	8	47	25.5	25.5	38.5	24.77
L cingulate cortex	32	28	-1.5	10.5	38.5	15.44
R paracentral lobule	4/6	37	7.5	-34.5	65.5	17.28
R inferior parietal lobule	40	29	37.5	-49.5	47.5	18.39
Valence^						
L middle frontal gyrus	47	43	-34.5	31.5	-3.5	25.03
L middle frontal gyrus	9	36	-40.5	19.5	26.5	16.12
R inferior frontal gyrus	9	138	34.5	7.5	-29.5	23.21
L amygdala		56	-19.5	-4.5	-9.5	25.33
R amygdala		45	19.5	-4.5	-12.5	25.89
L parahippocampal gyrus	36	77	-28.5	-40.5	-6.5	29.47
R parahippocampal gyrus	36	69	28.5	-40.5	-6.5	26.80
R sTG	22	38	61.5	-19.5	2.5	13.00
L/R culmen/ fusiform gyrus/ cuneus/ inferior occipital gyrus	17/37	3054	40.5	-40.5	-21.5	99.10
Task^						
L inferior frontal gyrus	47	392	-37.5	28.5	-3.5	57.92

© 2019 Blair KS et al. *JAMA Network Open.*

R inferior frontal gyrus	46	203	52.5	25.5	14.5	36.52
R medial frontal gyrus	9	129	4.5	43.5	32.5	15.31
L superior frontal gyrus	8	34	-19.5	28.5	50.5	14.64
L ACC	39	389	-4.5	43.5	-0.5	35.90
R precentral gyrus	4	109	28.5	-25.5	50.5	33.80
L precentral gyrus	6	34	-43.5	-13.5	35.5	32.31
L paracentral lobule	6	158	-4.5	-31.5	59.5	38.39
R amygdala/ parahippocampal gyrus		51	19.5	-4.5	-15.5	32.42
L amygdala/ parahippocampal gyrus		222	-28.5	-28.5	-12.5	31.48
L PCC	23	162	-4.5	-28.5	26.5	58.86
L PCC	29	32	-7.5	-49.5	11.5	19.51
L precuneus	39	236	-43.5	-70.5	35.5	29.03
R precuneus	31	83	7.5	-46.5	32.5	17.86
L mTG	21	189	-52.5	25.5	14.5	22.17
R mTG	21	182	58.5	-43.5	2.5	18.82
R mTG	21	83	52.5	-7.5	-9.5	19.13
L mTG	21	27	-61.5	-40.5	2.5	13.87
L/R cerebellum/ cuneus/ precuneus	7/17	188861	-28.5	-40.5	-27.5	99.60
Task-by-Sex						
L precentral gyrus	4	29	-19.5	-25.5	65.5	11.81

Activations are effects observed in whole brain analyses significant at p<0.001, corrected for multiple comparisons (significant at p<0.05), except ^p<0001 corrected for multiple comparisons (significant at p<0.05).

© 2019 Blair KS et al. *JAMA Network Open.*

eTable 3. Analysis of Significant Areas of Activation for Abuse Vs Neglect Not Reported in Table 2

REGION	BA	Voxels	X	Y	Z	F-value
Neglect						
R lingual gyrus	18	28	1.5	-76.5	2.5	14.91
Sex						
R paracentral lobule	5	28	1.5	-37.5	56.5	14.17
Abuse-by-Neglect						
L parahippocampal gyrus	36	56	-40.5	-31.5	-21.5	19.31
Valence^						
L middle frontal gyrus	47	44	-34.5	31.5	-3.5	24.51
L middle frontal gyrus	9	38	-37.5	19.5	26.5	16.86
R inferior frontal gyrus	46	117	52.5	31.5	11.5	25.72
R inferior frontal gyrus	47	20	28.5	28.5	-6.5	17.87
L amygdala/ parahippocampal gyrus		67	-19.5	-4.5	-9.5	23.24
R amygdala/ parahippocampal gyrus		71	31.5	-40.5	-3.5	24.99
L parahippocampal gyrus		60	-31.5	-40.5	-3.5	25.21
R sTG	21	26	55.5	-22.5	-0.5	12.62
L culmen/ fusiform gyrus/ cuneus/ inferior occipital gyrus		1170	-37.5	-40.5	-15.5	99.80
R culmen/ fusiform gyrus/ cuneus/ inferior occipital gyrus		1565	37.5	-40.5	-18.5	99.20

© 2019 Blair KS et al. *JAMA Network Open.*

Task^						
R middle frontal gyrus	9	240	40.5	34.5	26.5	23.93
L inferior frontal gyrus	47	620	-37.5	28.5	-3.5	48.47
R inferior frontal gyrus	46	166	52.5	25.5	14.5	32.61
R medial frontal gyrus	9	39	4.5	43.5	32.5	13.30
R precentral gyrus	4	113	28.5	-25.5	50.5	33.60
L PCC	31	104	-10.5	-43.5	35.5	22.52
L PCC	23	129	-4.5	-28.5	26.5	42.98
L PCC	39	28	-7.5	-49.5	11.5	19.22
L paracentral lobule	6	145	-4.5	-31.5	59.5	31.36
L precentral gyrus	6	31	-43.5	-13.5	35.5	25.88
R amygdala/ parahippocampal gyrus		38	19.5	-4.5	-15.5	21.45
L amygdala/ parahippocampal gyrus		218	-31.5	-31.5	-9.5	30.37
L mTG	21	123	-49.5	-4.5	-15.5	20.05
R sTG	39	142	43.5	-49.5	17.5	17.57
L sTG	39	237	-52.5	-61.5	29.5	21.83
R sTG	21	62	49.5	1.5	-12.5	21.09
L/R cerebellum/ cuneus/ precuneus	7/17	15647	34.5	-40.5	-30.5	100.00

Activations are effects observed in whole brain analyses significant at p<0.001, corrected for multiple comparisons (significant at p<0.05), except ^p<0001 corrected for multiple comparisons (significant at p<0.05).

© 2019 Blair KS et al. *JAMA Network Open.*

eTable 4. Analysis of Significant Areas of Activation for Total CTQ Score Reported in Table 2 With Added Covariates for Recruitment and Clinical Diagnoses

REGION	BA	Voxels	X	Y	Z	F-value
**CTQ Total Score**						
CTQ Total Score-by-Task Condition						
(A) Original Analysis						
R mid-cingulate cortex	31/6	51	7.5	-22.5	47.5	12.76
R postcentral gyrus	3	59	40.5	-25.5	56.5	9.81
L post/precentral gyrus/ premotor cortex gyrus	43	59	-52.5	-7.5	17.5	11.73
L mTG	21	35	-55.5	-13.5	-6.5	13.24
L sTG	22	26	-43.5	-25.5	-0.5	10.33
L declive/ culmen		76	-13.5	-58.5	-12.5	13.76
(B) With Added Community Covariate						
4R mid-cingulate cortex	31/6	37	7.5	-22.5	47.5	10.76
3R postcentral gyrus	3	40	49.5	-22.5	44.5	10.68
1L post/precentral gyrus/ premotor cortex gyrus	43	56	-55.5	-13.5	20.5	8.89
2L mTG	21	55	-55.5	-13.5	-6.5	10.61
9L sTG*	22	16	-43.5	-25.5	-0.5	6.23
8L declive/ culmen*		16	-13.5	-61.5	-185	7.07
(C) With Added CD Covariate						
14R mid-cingulate cortex	31/6	24	7.5	-22.5	47.5	8.51

© 2019 Blair KS et al. *JAMA Network Open.*

7R postcentral gyrus	3	48	52.5	-22.5	47.5	7.52
4L post/precentral gyrus/ premotor cortex gyrus	43	84	-52.5	-7.5	17.5	11.78
2L mTG/ sTG	21	150	-55.5	-13.5	-6.5	11.80
1L declive/ culmen		259	-13.5	-58.5	-9.5	10.68
(D) With Added ADHD Covariate						
6R mid-cingulate cortex	31/6	36	7.5	-22.5	47.5	10.52
5R postcentral gyrus	3	37	40.5	-25.5	56.5	7.65
2L post/precentral gyrus/ premotor cortex gyrus	43	74	-55.5	-13.5	20.5	10.96
1L mTG	21	82	-55.5	-13.5	-6.5	10.38
7L sTG*	22	20	-43.5	-25.5	2.5	7.79
3L declive/ culmen		69	-13.5	-58.5	-12.5	9.77
(E) With Added MDD Covariate						
5R mid-cingulate cortex	31/6	73	7.5	-22.5	47.5	12.04
1R postcentral gyrus	3	351	40.5	-25.5	56.5	12.95
3L post/precentral gyrus/ premotor cortex gyrus	43	75	-52.5	-7.5	17.5	9.03
7L mTG	21	52	-58.5	-13.5	-3.5	10.15
11L sTG	22	26	-43.5	25.5	2.5	8.26
2L declive/ culmen		341	-13.5	-58.5	-15.5	13.69
(F) With Added GAD Covariate						
10R mid-cingulate cortex*	31/6	19	7.5	-22.5	47.5	8.53
11R postcentral gyrus*	13	13	40.5	-25.5	56.5	7.82

© 2019 Blair KS et al. *JAMA Network Open.*

25L post/precentral gyrus/ premotor cortex gyrus*	43	9	-52.5	-7.5	17.5	7.17
4L mTG8*	21	19	-58.5	-13.5	-3.5	8.19
12L sTG8*	22	11	-43.5	25.5	2.5	7.23
1L declive/ culmen		36	-13.5	-58.5	-15.5	7.62
(G) With Added SAD Covariate						
6R mid-cingulate cortex	31/6	50	7.5	-22.5	47.5	8.69
1R postcentral gyrus	3	138	52.5	-19.5	44.5	9.89
5L post/precentral gyrus/ premotor cortex gyrus	43	67	-52.5	-7.5	17.5	11.77
8L mTG	21	30	-55.5	-13.5	-6.5	8.92
9L sTG	22	30	-40.5	-25.5	2.5	10.33
2L declive/ culmen		133	-25.5	-46.5	-21.5	8.84
(H) With Added PTSD Covariate						
6R mid-cingulate cortex	31/6	31	7.5	-22.5	47.5	8.25
9R postcentral gyrus*	3	19	40.5	-28.5	56.5	7.47
3L post/precentral gyrus/ premotor cortex gyrus	43	83	-52.5	-7.5	17.5	11.23
1L mTG/ sTG	21	114	-55.5	-13.5	-6.5	11.13
2L declive/ culmen		96	-13.5	-58.5	-15.5	10.30

(**A**) Original data reported in Table 2, significant at p<0.001, corrected for multiple comparisons (significant at p<0.05). (**B** to **H**) Added covariate involving (**B**) recruitment (community v. residential care); (**C**) CD (present v. not present); (**D**) ADHD (present v. not present); (**E**) MDD (present v. not present); (**F**) GAD (present v. not present); (**G**) SAD (present v. not present); and (**H**) PTSD (present v. not present). Activations are from whole brain analyses significant at

© 2019 Blair KS et al. *JAMA Network Open.*

p<0.005, corrected for multiple comparisons significant at p<0.05 (except * not corrected for multiple comparisons).

eTable 5. Analysis of Significant Areas of Activation for Abuse vs Neglect Reported in Table 2 With Added Covariates for Recruitment and Clinical Diagnoses

**REGION**	**BA**	**Voxels**	**X**	**Y**	**Z**	**F-value**
**Abuse vs. Neglect Score**						
(A) Original Analysis						
Abuse-by-Task Condition						
L rmPFC	9	54	-16.5	49.5	26.5	10.36
R mid-cingulate cortex	31/6	52	7.5	-22.5	47.5	13.96
R postcentral gyrus/ inferior parietal lobule	40	235	31.5	-37.5	53.5	15.21
L pre/postcentral gyrus/ premotor cortex	6	109	-49.5	-7.5	23.5	11.16
R pre/postcentral gyrus	3	30	61.5	-10.5	23.5	13.30
Neglect-by-Task Condition						
R cuneus	18	62	7.5	-76.5	17.5	10.28
(C) With Added CD Covariate						
Abuse-by-Task Condition						
2L/R rmPFC/ACC	9/24	550	10.5	28.5	-6.5	10.40
7R mid-cingulate cortex	24/31/6	69	10.5	-19.5	44.5	12.92
3R postcentral gyrus/ inferior parietal lobule	40	283	31.5	-37.5	53.5	12.69
4L pre/postcentral gyrus/ premotor cortex	6/3	153	-55.5	-10.5	20.5	11.67
3R pre/postcentral gyrus	3/40	283	31.5	-37.5	53.5	12.72
Neglect-by-Task Condition						

© 2019 Blair KS et al. *JAMA Network Open.*

R cuneus	18	62	7.5	-76.5	17.5	10.28
(D) With Added ADHD Covariate						
Abuse-by-Task Condition						
L rmPFC						
R mid-cingulate cortex	31/6	52	7.5	-22.5	47.5	13.96
R postcentral gyrus/ inferior parietal lobule	40	235	31.5	-37.5	53.5	15.21
L pre/postcentral gyrus/ premotor cortex	6	109	-49.5	-7.5	23.5	11.16
R pre/postcentral gyrus	3	30	61.5	-10.5	23.5	13.30
Neglect-by-Task Condition						
R cuneus	18	62	7.5	-76.5	17.5	10.28
(E) With Added MDD Covariate						
Abuse-by-Task Condition						
L rmPFC						
R mid-cingulate cortex	31/6	52	7.5	-22.5	47.5	13.96
R postcentral gyrus/ inferior parietal lobule	40	235	31.5	-37.5	53.5	15.21
L pre/postcentral gyrus/ premotor cortex	6	109	-49.5	-7.5	23.5	11.16
R pre/postcentral gyrus*	3	30	61.5	-10.5	23.5	13.30
(F) With Added GAD Covariate						
Abuse-by-Task Condition						
2L rmPFC	9	93	-10.5	46.5	20.5	8.81
5R mid-cingulate cortex	31/6	28	7.5	-22.5	47.5	9.99
1R postcentral gyrus/ 2L inferior parietal lobule	40	163	-52.5	-19.5	41.5	11.03

© 2019 Blair KS et al. *JAMA Network Open.*

L pre/postcentral gyrus*	6	12	-55.5	-10.5	20.5	6.72
Neglect-by-Task Condition						
R cuneus	18	309	16.5	-88.5	26.5	11.05
(G) With Added SAD Covariate						
Abuse-by-Task Condition						
1L rmPFC	9/10	188	-10.5	46.5	17.5	12.72
3R mid-cingulate cortex	31/6	106	19.5	-10.5	53.5	14.74
2R postcentral gyrus/ inferior parietal lobul	40	130	31.5	-37.5	50.5	10.20
8L pre/postcentral gyrus/ premotor cortex/ insula	6	68	-34.5	-7.5	14.5	9.90
81R pre/postcentral gyrus*	3	2	58.5	-10.5	23.5	5.79
Neglect-by-Task Condition						
R cuneus/ posterior cingulate cortex	18	257	13.5	-49.5	11.5	9.23
(H) With Added PTSD Covariate						
Abuse-by-Task Condition						
41L rmPFC*	9	3	-16.5	49.5	26.5	5.78
7R mid-cingulate cortex*	31/6	15	10.5	-22.5	44.5	8.24
1R postcentral gyrus/ inferior parietal lobule	40/3	148	49.5	-19.5	38.5	11.19
2L pre/postcentral gyrus/ premotor cortex/ insula	6	40	-37.5	-7.5	14.5	8.61
16R pre/postcentral gyrus/ insula*	3/13	7	46.5	-7.5	14.5	13.30
Neglect-by-Task Condition						
R cuneus	18	547	-4.5	-88.5	20.5	13.16

© 2019 Blair KS et al. *JAMA Network Open.*

(**A**) Original data reported in Table 2, significant at p<0.001, corrected for multiple comparisons (significant at p<0.05). (**B** to **H**) Added covariate involving (**B**) recruitment (community v. residential care); (**C**) CD (present v. not present); (**D**) ADHD (present v. not present); (**E**) MDD (present v. not present); (**F**) GAD (present v. not present); (**G**) SAD (present v. not present); and (**H**) PTSD (present v. not present). Activations are from whole brain analyses significant at p<0.005, corrected for multiple comparisons significant at p<0.05 (except * not corrected for multiple comparisons).

© 2019 Blair KS et al. *JAMA Network Open.*

eTable 6. Analysis of Significant Areas of Activation for Abuse: EA vs PA

REGION	BA	Voxels	X	Y	Z	F-value
EA-by-Task						
R inferior parietal lobule	40	40	31.5	-37.5	53.5	13.68
L culmen/declive		24	-13.5	-58.5	-12.5	9.88
PA-by-Valence						
R dorsolateral frontal gyrus	8/9	72	31.5	40.5	32.5	14.50
R dmPFC	6/8	23	7.5	31.5	56.5	9.91
Sex						
R middle frontal gyrus	8	34	25.5	25.5	38.5	24.97
L paracentral lobule	4	29	-1.5	-34.5	62.5	15.72
Valence^						
R middle frontal gyrus	9	42	37.5	10.5	29.5	15.64
L middle frontal gyrus		41	-34.5	31.5	-3.5	23.39
L middle frontal gyrus	9	24	-40.5	19.5	26.5	13.83
R inferior frontal gyrus	46	27	5.5	28.5	14.5	18.77
R amygdala/ parahippocampal gyrus		31	19.5	-4.5	-12.5	20.85
R parahippocampal gyrus	36	64	28.5	-40.5	-15.5	25.48
L parahippocampal gyrus	36	51	-25.5	-43.5	-6.5	28.22
R sTG	21	31	55.5	-22.5	2.5	13.87
L uncus		45	-34.5	-7.5	-24.5	18.31
L culmen/ fusiform gyrus/ cuneus/ inferior occipital gyrus		1001	-37.5	-40.5	-15.5	100.00

© 2019 Blair KS et al. *JAMA Network Open.*

R culmen/ fusiform gyrus/ cuneus/ inferior occipital gyrus		1439	40.5	-40.5	-18.5	100.00
Task^						
R middle frontal gyrus	9	271	40.5	34.5	26.5	26.58
R inferior frontal gyrus	46	145	52.5	25.5	14.5	29.09
L inferior frontal gyrus	47	338	-37.5	28.5	-3.5	48.91
L superior frontal gyrus	8	21	-16.5	34.5	50.5	13.06
L medial frontal gyrus	8	111	-1.5	40.5	38.5	18.45
L ACC	32	341	-1.5	34.5	-3.5	32.41
R precentral gyrus	4	118	28.5	-25.5	50.5	33.36
L precentral gyrus	6	29	-43/5	-13.5	35.5	23.12
R paracentral lobule	6	137	7.5	-22.5	47.5	31.75
L PCC	29	25	-7.5	-49.5	11.5	19.54
R amygdala/ parahippocampal gyrus		49	22.5	-7.5	-12.5	15.10
L parahippocampal gyrus		213	-31.5	-31.5	-6.5	31.49
R precuneus	31	75	7.5	-46.5	32.5	16.25
L mTG	21	180	-49.5	-4.5	-15.5	21.64
R mTG	21	43	55.5	1.5	-12.5	15.10
L sTG	39	205	-52.5	25.5	14.5	24.21
R sTG	39	162	43.5	-49.5	17.5	19.52
L/R cerebellum/ cuneus/ precuneus	7/17	16775	31.5	-40.5	-30.5	100.00

Activations are effects observed in whole brain analyses significant at $p<0.001$, corrected for multiple comparisons (significant at $p<0.05$), except ^p<0001 corrected for multiple comparisons (significant at $p<0.05$).

© 2019 Blair KS et al. *JAMA Network Open.*

eTable 7. Analysis of Significant Areas of Activation for SA: SA vs EA, PA, EN, and PN

REGION	BA	Voxels	X	Y	Z	F-value
SA-by-Task						
R ACC/rmPFC	24	510	10.5	31.5	2.5	15.74
R aIC	13	55	34.5	7.5	5.5	12.87
L aIC	13	32	-31.5	4.5	8.5	10.76
R putamen		37	13.5	7.5	-0.5	11.42
R postcentral gyrus/ inferior parietal cortex	2	115	43.5	-22.5	44.5	16.08
L post/precentral gyrus	6	41	-43.5	-16.5	32.5	12.47
R precuneus/PCC	31	24	16.5	-58.5	20.5	10.80
SA-by-Valence-by-EA/PA/EN/PN						
R culmen		34	7.5	-58.5	-6.5	11.21
L PCC	31	43	-16.5	-64.5	14.5	10.19
Valence^						
R inferior frontal gyrus		55	34.5	1.5	32.5	17.47
L parahippocampal gyrus		34	-31.5	-40.5	-3.5	19.05
R parahippocampal gyrus		86	28.5	-46.5	-6.5	26.99
L culmen/ fusiform gyrus/ cuneus/ iFG		484	-37.5	-43.5	-12.5	46.15
R culmen/ fusiform gyrus/ cuneus/ iFG		767	49.5	-67.6	-0.5	55.58
SA-by-Valence-by-EA/PA/EN/PN						
R culmen		65	7.5	-58.5	-6.5	11.21

© 2019 Blair KS et al. *JAMA Network Open.*

L PCC	31	56	-16.5	-64.5	14.5	10.19
Task^						
L middle frontal gyrus	9	84	-34.5	34.5	29.5	27.43
L inferior frontal gyrus	45	185	-49.5	25.5	8.5	46.54
L ACC	24	158	-4.5	25.5	3.5	23.57
L/R postcentral gyrus/ ACC/ precentral gyrus		2970	-49.5	-22.5	47.5	100.00
L paracentral lobule	6	42	-4.5	-28.5	65.5	22.33
R postcentral gyrus	3	21	55.5	-16.5	26.5	23.32
L ACC	31	71	-7.5	-40.5	35.5	23.37
L/R thalamus/ putamen/ caudate		1858	-10.5	-16.5	11.5	98.79
L parahippocampal gyrus	36	48	-22.5	-34.5	-9.5	25.12
L mTG	21	34	-61.5	-13.5	-6.5	19.77
R inferior parietal lobule	40	418	40.5	-34.5	41.5	44.41
L angular gyrus	39	106	-49.5	-64.5	32.5	23.62
L middle occipital gyrus	19/18	128	-34.5	-79.5	-6.5	30.62
R inferior occipital gyrus	19	51	40.5	-79.5	-3.5	20.69
L cuneus		173	-10.5	-67.5	8.5	30.08
L culmen/ fusiform gyrus		204	-28.5	-46.5	-24.5	46.57
R culmen/ fusiform gyrus		918	25.5	-46.5	-21.5	99.20

Activations are effects observed in whole brain analyses significant at p<0.001, corrected for multiple comparisons (significant at p<0.05), except ^p<0001 corrected for multiple comparisons (significant at p<0.05).

© 2019 Blair KS et al. *JAMA Network Open.*

eTable 8. Analysis of Significant Areas of Activation for Neglect: EN vs PN

REGION	BA	Voxels	X	Y	Z	F-value
Sex						
L precuneus	31/7	26	-13.5	-70.5	29.5	17.46
EN-by-Task Condition-by-Valence						
R superior frontal gyrus		33	1.5	34.5	44.5	7.51
R superior frontal gyrus		25	43.5	-67.5	32.5	8.02
Valence^						
R middle frontal gyrus	46	138	52.5	31.5	14.5	22.27
L middle frontal gyrus		60	-34.5	31.5	-3.5	22.41
L middle frontal gyrus		46	-37.5	19.5	26.5	19.24
R inferior frontal gyrus	47	36	28.5	28.5	-6.5	22.04
L amygdala		67	-19.5	-4.5	-9.5	25.82
L parahippocampal gyrus	36	62	-28.5	-40.5	-6.5	23.65
R parahippocampal gyrus	36	58	28.5	-40.5	-6.5	24.45
R sTG	21	30	64.5	-22.5	-0.5	12.16
L culmen/ fusiform gyrus/ cuneus/ inferior occipital gyrus/amygdala		1223	-37.5	-37.5	-15.5	100.00
R culmen/ fusiform gyrus/ cuneus/ inferior occipital gyrus/amygdala		1630	40.5	-40.5	-21.5	100.00
Task^						

© 2019 Blair KS et al. *JAMA Network Open.*

L inferior frontal gyrus		319	-40.5	28.5	-3.5	46.07
R inferior frontal gyrus	46	159	52.5	25.5	14.5	31.54
R middle frontal gyrus	9	264	37.5	31.5	32.5	25.73
L superior frontal gyrus	6	26	-19.5	25.5	53.5	13.37
L ACC	32	290	-4.5	34.5	-3.5	29.79
R precentral gyrus	4	106	28.5	-25.5	50.5	31.40
L precentral gyrus		36	-40.5	-13.5	35.5	27.60
L paracentral lobule		149	-4.5	-31.5	59.5	33.52
L PCC	23	131	-4.5	-28.5	26.5	37.19
L PCC	31	93	-7.5	-40.5	38.5	20.86
L PCC		28	-7.5	-49.5	11.5	18.07
R amygdala/ parahippocampal gyrus		50	19.5	-4.5	-15.5	27.87
L amygdala/ parahippocampal gyrus		224	-31.5	-31.5	-9.5	35.66
L mTG		162	-49.5	-4.5	-15.5	22.56
R mTG	21	108	58.5	-43.5	2.5	15.21
L sTG	39	236	-52.5	-61.5	29.5	22.64
R sTG	21	73	49.5	1.5	-12.5	20.96
L/R cerebellum/ cuneus/ precuneus		16310	31.5	-43.5	-30.5	100.00

© 2019 Blair KS et al. *JAMA Network Open.*

Activations are effects observed in whole brain analyses significant at $p<0.001$, corrected for multiple comparisons (significant at $p<0.05$), except ^$p<.0001$ corrected for multiple comparisons (significant at $p<0.05$).

© 2019 Blair KS et al. *JAMA Network Open.*

eTable 9. Analysis of Significant Areas of Activation for Abuse as the Only Covariate

REGION	BA	Voxels	X	Y	Z	F-value
Abuse-by-Task Condition						
L rmPFC^^	9	27	-13.5	49.5	23.5	10.30
R mid-cingulate cortex	31/6	58	7.5	-22.5	47.5	15.24
R post/precentral gyrus/ premotor cortex gyrus	2	235	52.5	-19.5	44.5	13.60
L post/precentral gyrus/ premotor cortex gyrus	43	134	-55.5	-10.5	20.5	15.71
L mTG	21	-55.5	-13.5	-13.5	-6.5	12.03
L sTG	22	43	-43.5	-37.5	2.5	13.38
L culmen		48	-22.5	-46.5	-21.5	11.11
R insula	13	27	46.5	-7.5	14.5	13.23
Sex						
R middle frontal gyrus	8	54	25.5	25.5	38.5	25.63
L dmFC	32	24	-1.5	10.5	38.5	16.09
L pre/postcentral gyrus/	6	109	-49.5	-7.5	23.5	11.16
R inferior parietal lobule	40	24	37.5	-49.5	47.5	17.45
Valence^						
L middle frontal gyrus		57	-34.5	31.5	-3.5	27.60
L middle frontal gyrus	9	47	-40.5	19.5	26.5	16.75
R inferior frontal gyrus	9	164	34.5	7.5	29.5	24.57
R inferior frontal gyrus	47	24	28.5	28.5	-6.5	19.42

© 2019 Blair KS et al. *JAMA Network Open.*

L amygdala		74	-19.5	-4.5	-9.5	27.67
L parahippocampal gyrus		83	-31.5	-40.5	-3.5	32.00
R parahippocampal gyrus		76	28.5	-40.5	-6.5	28.49
R sTG	22	38	64.5	-19.5	2.5	13.66
L/R culmen/ fusiform gyrus/ cuneus/ inferior occipital gyrus/amygdala		3286	40.5	-40.5	-21.5	100.00
Task^						
R inferior frontal gyrus	46	203	52.5	25.5	14.5	35.80
L inferior frontal gyrus	47	411	-37.5	28.5	-3.5	61.22
R medial frontal gyrus	9	203	4.5	43.5	32.5	16.30
L ACC	32	407	-4.5	43.5	-0.5	37.81
L precentral gyrus	6	47	-43.5	-13.5	35.5	34.25
L paracentral lobule	6	163	-4.5	-31.5	59.5	38.01
L PCC	23	165	-4.5	-28.5	26.5	62.18
L PCC	29	36	-7.5	-49.5	11.5	22.80
R amygdala/ parahippocampal gyrus		60	19.5	-4.5	-15.5	36.93
L amygdala/ parahippocampal gyrus		242	-28.5	-28.5	-12.5	33.10
R precuneus	31	104	7.5	-46.5	32.5	19.15
L mTG	22	36	-52.5	-40.5	2.5	14.29

© 2019 Blair KS et al. *JAMA Network Open.*

L mTG	21	220	-49.5	-4.5	-15.5	24.88
R mTG	21	207	58.5	-43.5	2.5	20.22
R mTG	21	106	55.5	-7.5	-9.5	21.11
L sTG	39	249	-52.5	-61.5	29.5	28.55
L/R cerebellum/ cuneus/ precuneus		19498	-28.5	-40.5	-27.5	100.00

Activations are effects observed in whole brain analyses significant at p<0.001, corrected for multiple comparisons (significant at p<0.05), except ^p<0001 and ^^p<002 corrected for multiple comparisons (significant at p<0.05).

© 2019 Blair KS et al. *JAMA Network Open.*

eTable10. Analysis of Significant Areas of Activation for Neglect as the Only Covariate

**REGION**	**BA**	**Voxels**	**X**	**Y**	**Z**	**F-value**
Neglect-by-Task Condition						
L middle temporal gyrus	21	32	-55.5	-13.5	-6.5	13.02
Sex						
R middle frontal gyrus	8	54	25.5	25.5	38.5	25.89
R paracentral lobule	4	31	7.5	-34.5	62.5	17.11
L precuneus	31	24	-10.5	-70.5	26.5	18.82
Valence^						
L middle frontal gyrus		58	-34.5	31.5	-3.5	27.54
L middle frontal gyrus	9	47	-40.5	19.5	26.5	16.85
R inferior frontal gyrus		167	34.5	7.5	-29.5	24.15
R inferior frontal gyrus	47	24	28.5	28.5	-6.5	19.89
L amygdala/parahippocampal gyrus		73	-19.5	-4.5	-9.5	27.59
R parahippocampal gyrus	36	76	28.5	-40.5	-6.5	28.39
L parahippocamal gyrus	19	82	-31.5	-40.5	-3.5	31.61
R sTG	22	38	61.5	-19.5	2.5	13.66
L/R culmen/ fusiform gyrus/ cuneus/ inferior occipital gyrus/ amygdala		3286	40.5	-40.5	-21.5	100.00
Task^						
L inferior frontal gyrus		408	-40.5	28.5	-3.5	59.35
R inferior frontal gyrus	46	202	52.5	25.5	14.5	35.48
R medial frontal gyrus	9	148	4.5	43.5	32.5	15.71

© 2019 Blair KS et al. *JAMA Network Open.*

L superior frontal gyrus	8	35	-16.5	34.5	50.5	14.60
L ACC	32	410	-4.5	43.5	-0.5	37.46
L PCC	23	166	-4.5	-28.5	26.5	63.10
L PCC	29	36	-7.5	-49.5	11.5	22.32
R amygdala/ parahippocampal gyrus		59	19.5	-4.5	-15.5	36.92
L parahippocampal gyrus/ amgydala		238	-28.5	-28.5	-12.5	32.38
R precentral gyrus	4	118	28.5	-25.5	50.5	35.74
L precentral gyrus	6	45	-40.5	-13.5	35.5	34.15
L paracentral lobule	6	163	-4.5	-31.5	59.5	38.10
R precuneus		105	7.5	-46.5	32.5	19.34
L sTG	39	247	-52.5	-61.5	29.5	28.40
R mTG	21	106	55.5	-7.5	-9.5	20.99
L mTG	21	222	-49.5	-4.5	-15.5	23.84
R mTG	21	210	58.5	-43.5	2.5	20.25
L mTG	21	34	-61.5	-40.5	2.5	14.21
L/R cerebellum/cuneus/precuneus		19540	-28.5	-40.5	-27.5	100.00
Neglect-by-Task Condition-by-Sex						
L precuneus		30	-22.5	-52.5	53.5	10.49
R cuneus	18	28	13.5	-70.5	17.5	13.66

Activations are effects observed in whole brain analyses significant at $p<0.001$, corrected for multiple comparisons (significant at $p<0.05$), except ^p<0001 corrected for multiple comparisons (significant at $p<0.05$).

© 2019 Blair KS et al. *JAMA Network Open.*
